# Characterization of Google Search Volumes and Trends From 2004 to 2021 for Diagnosis and Treatment of Locally Advanced Prostate Cancer

**DOI:** 10.7759/cureus.42725

**Published:** 2023-07-31

**Authors:** Jessica Kim, Anthony Brooks, Zachariah Taylor, Paulette Dreher, Gregory C McMahon

**Affiliations:** 1 Emergency Medicine, Philadelphia College of Osteopathic Medicine, Philadelphia, USA; 2 Urology, Main Line Health, Philadelphia, USA; 3 Urology, MidLantic Urology, Wynnewood, USA

**Keywords:** quality of life, side effects, prostatectomy, psa, internet, prostate cancer

## Abstract

Introduction and objective

The purpose of this study is to better characterize at which point during the course of diagnosis and treatment of locally advanced prostate cancer the internet is utilized and to evaluate the search trends over time.

Methods

Monthly Google Trends data were collected from 2004 to 2021 for prostate cancer-specific terms. Temporal trends were analyzed by comparing average search volume indexes (aSVI) and analysis with joinpoint software of six-month percent change (6mPC). Chloropleths were created for geographic pattern comparisons.

Results

Search terms associated with interventions demonstrated the highest aSVI with terms such as “prostate biopsy” (aSVI: 33.59), “prostatectomy” (aSVI: 31.6), and “prostate radiation” (aSVI: 16.45). Terms associated with treatment side effects increased at a high rate with “radiation side effects” (21.4 6mPC, p<0.05) and “prostatectomy side effects” (14.4 6mPC, p<0.05). Prostate-specific antigen (PSA)-related search terms demonstrated a strong positive trend on joinpoint analysis with search terms “What is PSA?” (8.9 6mPC, p<0.05), and “What is normal PSA?” (15.1, p<0.05). Geographic patterns demonstrated higher search volumes in regard to screening and diagnostic terms in the northeast, while the southern regions demonstrated relatively higher search volumes for treatment and interventions.

Conclusions

The internet continues to be a growing part of the dynamics of prostate cancer management with more men utilizing the internet each year to help understand their diagnosis. Specifically, we found that the internet is used more for searches pertaining to PSA, procedures, and interventions affecting the quality of life.

## Introduction

Prostate cancer (PCa) is the most commonly diagnosed cancer in men with 191,930 cases diagnosed in 2020 which represents 10.6% of all new cancer cases [1]. Traditionally, screening for prostate cancer includes a digital rectal exam (DRE) and prostate-specific antigen (PSA), with prostate biopsy used for tissue confirmation of the diagnosis and for stratifying men into risk groups. Treatment indications and options for men with prostate cancer continue to be an evolving topic. Treatment options for clinically localized prostate cancer have now expanded to include active surveillance as the preferred management strategy, minimally invasive robotic surgery, various types of radiotherapy, hormone therapy, chemotherapy, and clinical trials that offer novel developments [2].

Resources for prostate cancer are readily available online and offer a range of information from risk, screening, diagnosis, and treatment to statistics, data, health tips, and support groups [3]. These brochures are designed and tailored to patients stratified by stages of prostate cancer, and even provide information on genetic testing to help patients better prepare on how they should approach their disease. These sources are made to help patients make informed choices regarding their treatment and to provide a better outlook on life post-treatment and how they can cope with their disease.

Despite provider counseling, it is not surprising that individuals often feel the need to turn to the internet for additional information. Information-seeking behavior is often employed as a technique for coping with illness and can affect anxiety, attitude, and quality of life (QoL) [4,5]. With the dawn of the internet and the availability of instant information, the internet has become the primary source of information and specifically, medical information for the general public and cancer patients alike [6,7]. Many prostate cancer educational efforts, by use of interactive computer programs and websites, have been shown to be helpful to patients [8]. Patients overall felt well-informed, empowered, and able to better deal with their disease, while some studies even reported reduced symptoms of depression [8]. However, it remains unclear when during the course of their disease that patients, caregivers, and/or family members seek clarity and additional information from the internet, and how its use may have changed over time with the transformation of prostate cancer management.

In this study, we aim to better characterize internet usage and trends along the diagnosis and treatment course of the diagnosis and treatment of locally advanced prostate cancer.

## Materials and methods

Monthly Google Trends data were collected from 2004-2021 at the state and national levels offering both geographic and temporal data patterns. Search terms were selected to adequately represent the interest in both the diagnostic and treatment phases of the prostate cancer pathway. Search terms were selected to represent terms that patients would commonly use. The following English terms were used: “prostate cancer”, “prostate cancer survival”, “PSA”, “What is PSA?”, “What is normal PSA?”, “prostate biopsy”, “Gleason score”, “active surveillance”, “prostatectomy”, “prostatectomy side effects”, “prostate radiation”, and “prostate radiation side effects”. To better compare search terms, they were grouped into the following groups: general knowledge, diagnosis, and treatment. The search volume index (SVI) for each term was compared to the highest SVI in that group and all other SVI in that group are relative to that term. The SVI was generated from the Google Trends website over the duration of the time period. SVI scores are relative to the highest point on the chart for the given region and time period and do not represent absolute search volume numbers. A value of 100 is the peak search volume for the term, and a value of 50 would equal half of the peak search volume. SVI for each term was then averaged (aSVI). Choropleths of state-specific aSVI for the entire study period were made to demonstrate the geographic distribution of search volume across the United States.

Joinpoint analysis was performed using the National Cancer Institute’s Joinpoint calculator to better understand changes in the trend throughout the study period [9]. For the data to be analyzed by Joinpoint, the monthly SVI for each term per year was grouped into six-month averages to calculate the percent change per six-month period (6mPC) over the given time period. The 6mPC was then compared via a linear regression model, a negative 6mPC is indicative of declining interest, while a positive one was indicative of increasing interest.

All statistical analyses were performed using R (R Foundation for Statistical Computing, Vienna, Austria) and Joinpoint Trend Analysis Software V. 4.2.0.2 (Statistical Research and Applications Branch, National Cancer Institute, Bethesda, MD, USA).

## Results

Google Trends 

**Figure 1:**
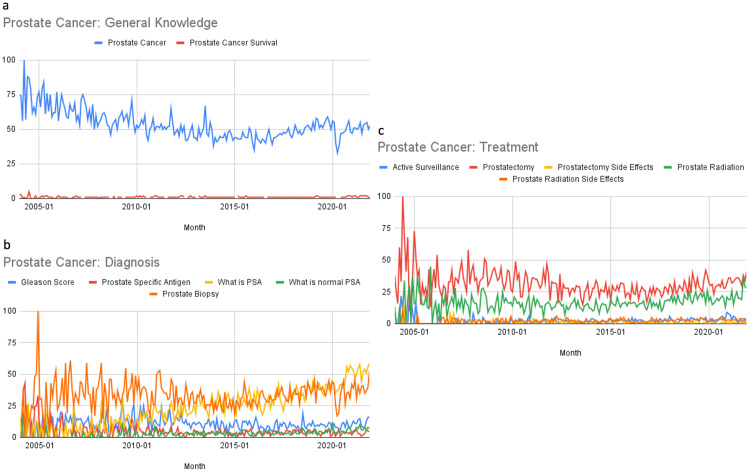
Monthly search volume index (SVI) from 2004 to 2021 for each term. Terms were organized into three distinct groups for comparison (a) general knowledge, (b) diagnosis, (c) treatment. PSA: prostate-specific antigen

Monthly SVI was calculated for each search term from January 2004 to September 2021. Terms were organized into three distinct groups for comparison (general knowledge, diagnosis, treatment). SVI for each group is represented for the duration of the study period (Figure 1). 

In the general knowledge group, the term “prostate cancer” demonstrated a higher SVI than the term “prostate cancer survival”. The month with the greatest SVI for “prostate cancer” was April 2004, while it was July 2004 for “prostate cancer survival”. 

In the diagnosis group, “prostate biopsy” demonstrated the highest average SVI with an average of 33.59 with a peak in December 2004. “What is PSA” demonstrated the next highest average SVI at 23.55 (peak of 59 in September 2021), then “Gleason score” at 10.59 (peak 31 in September 2009), “prostate-specific antigen” at 5.125 (peak of 43 in April 2004), and “what is normal PSA” at 2.93 (March 2004).

In the prostate cancer treatment group, “prostatectomy” demonstrated the highest aSVI at 31.60 (peak: 100, June 2004). “Prostate radiation” followed next with an aSVI of 16.45 (peak: 45, November 2005). A significant reduction in aSVI was observed with “active surveillance” with an aSVI of 2.56 (peak: 22, May 2004), followed closely by “prostate radiation side effects” with an aSVI of 2.19 (peak: 11, June 2004).

**Figure 2:**
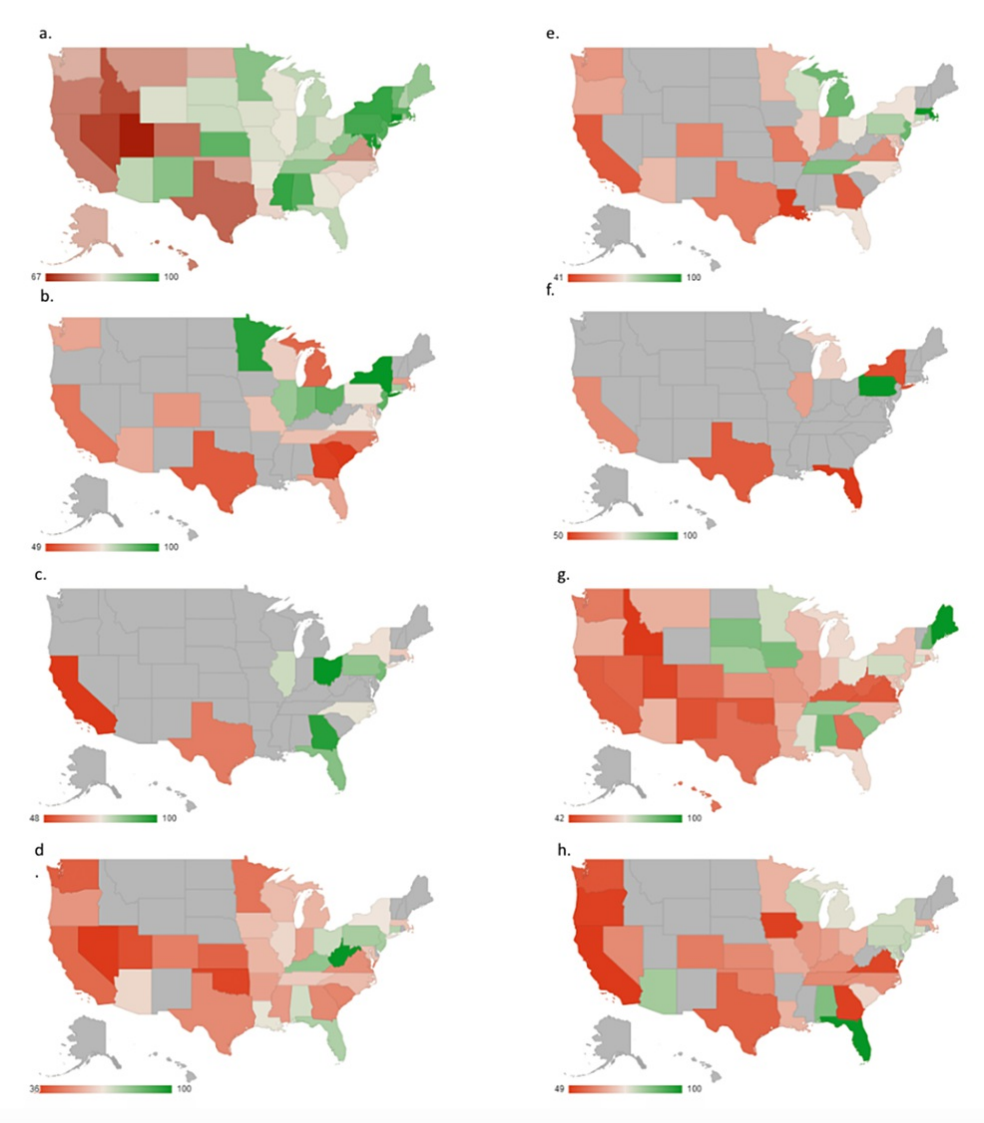
Geographical distribution of average search volume index (aSVI) with green representative of higher aSVI and red of lower aSVI. Grey states did not have sufficient SVI for inclusion. a) prostate cancer. b) prostate cancer survival. c) Gleason score. d) PSA. e) What is normal PSA? f) prostate biopsy. g) prostatectomy. h) prostate radiation. PSA: prostate-specific antigen

Overall, the Northeastern states (New York, Pennsylvania, New Jersey, Connecticut, Massachusetts, etc.) demonstrated higher aSVI than states on the West Coast, South, or Midwest for most search terms (Figure 2). There are however a few notable exceptions. Prostatectomy demonstrated a higher SVI in the Midwest states (South Dakota, Nebraska, Minnesota, Iowa) and Southern states (Tennessee, Alabama, South Carolina) than in the Northeast or Western states (Figure 2G). However, Maine demonstrated the highest SVI for prostatectomy. Search terms in the prostate cancer screening and diagnosis group demonstrated higher SVI in the Northeast states, while treatment-focused searches (prostatectomy, prostate radiation side effects, and prostate biopsy) demonstrated relatively higher SVI in the Southern states. 

Trend analysis by Joinpoint

To better characterize changes in trends throughout the study period, Joinpoint analysis was applied to the six-month average SVI for each term (Table 1). All but two of the terms demonstrated a positive slope over the course of the study period and are shown in Table 1 in descending order: Radiation side effects (21.1), What is normal PSA (15.1*), Prostatectomy side effects (14.4*), What is PSA (8.9*), Gleason Score (2.2*), Prostate Cancer Survival (1.9), Prostate-specific antigen (0.8*), and Prostate Biopsy (0.7*). Only “prostate cancer” (-0.6*), and “prostatectomy” (-0.2*) demonstrated a negative slope for the duration of the study period.

**Table 1:** Trend analysis by Joinpoint * Indicates that the 6-Month Average Percent Change (6mPC) is significantly different from zero at the alpha = 0.05 level. - The statistics could not be calculated. ˣ, ⁰ Indicates that the respective variables were compared to each other when determining SVI. PSA: prostate-specific antigen, SVI: search volume index

Variable	United States 2004-2021		Trend 1			Trend 2			Trend 3			Trend 4			
	Avg. 6mPC	CI	Period	6mPC	CI	Period	6mPC	CI	Period	6mPC	CI	Period	6mPC	CI	
Prostate Cancer	-0.6*	-10, -0.3	1-23	-1.7*	-2.1, -1.4	24-36	1.2*	0.4, 2.1							
Prostate Cancer Survival	1.9	-3.3, 7.4	1-10	-1.5	-6.6, 3.8	10-13	25.3	25.3, 123.9	13-29	-2.9*	-5.3, -0.5	29-36	8.7*	0.6, 17.5	
Gleason Score	2.2*	0.3, 4.2	1-3	59.4*	13.9, 123.1	3-36	-0.5*	2.3-4.1							
Prostate-specific Antigen	0.8*	0.4, 1.3	1-22	-0.7*	-1.2, -0.2	22-36	3.2*	2.3-4.1							
“What is PSA?”	8.9*	6.2, 11.7	1-36	8.9*	6.2, 11.7										
“What is Normal PSA?”	15.1*	6.5, 24.3	1-3	999.8*	437.8, 2149.0	3-8	-53.0*	-62.5, -41.1	8-11	196.2*	44.9, 505.8	11-36	2.6*	1.2, 4.1	
Prostate Biopsy	0.7*	0.2, 1.2	1-36	0.7*	0.2, 1.2										
Active Surveillance	10	-0.5, 21.7	1-5	-5.4	-33.7, 34.8	5-8	195.6	-3.7, 807.2	8-36	1.1					
Prostatectomy^x^	-0.2	-0.8, 0.5	1-25	-1.7*	-2.2, -1.1	25-36	3.3*	1.4, 3.5							
Prostate Radiation^x^	1.22*	0.6, 1.9	1-36	1.22*	0.6, 1.9										
Prostatectomy Side Effects^0^	14.4*	9.5, 19.6	1-36	14.4*	9.5, 19.6										
Radiation Side Effects^0^	21.4*	13.1, 29.1	1-5	291.1*	130.6, 566	5-36	4.4*	1.9, 6.9							

The term “prostate cancer” demonstrated two trends within the time period, with the first trend from January 2004 to December 2015 with a 6mPC of -1.7* (-2.1, -1.4), and then changing to a positive trend starting from January 2016 with a 6mPC of 1.2* (0.4-2.1) until the time of data collection. “Prostate cancer survival” demonstrated four trends with the first from January 2004 to June 2007 as a nonsignificant trend of -1.5 (-6.6, 3.8), and then a significant 6mPC of 25.3* from July 2008 to December 2010 (15.3, 123.9), followed by a significant 6mPC of -2.9* from January 2010 to June 2018 (-5.3, -0.5), and then finally an upward trend of 8.7 from January 2018 to the time of data collection (0.6, 17.5).

Terms in the diagnosis group generally had positive trends throughout the time period. “Gleason score” demonstrated two trends with a strongly positive 6mPC from January 2004 to June 2005 at 59.4* (13.9, 123.1), and then a second trend from July 2005 to the time of data collection 6mPC of -0.5* (-0.9, -0.0). “Prostate-specific antigen” demonstrated two trends with the first from January 2004 to December 2014 at -0.7* (-1.2, -0.2), and then at 3.2* (2.3-4.1) from segment January 2015 to time of data collection. “What is PSA?” demonstrated only one trend, 8.9* (6.2, 11.7) during the entire time period. “What is normal PSA?” however, demonstrated four trends with the first from January 2004 to June 2005 with a very sharp 6mPC of 999.8* (437.8, 2149.0), which then shifted to a negative 6mPC of -53.0 (-62.5, -41.1) from July 2005 to June 2007. It again then changed to a strongly positive 6mPC from July 2007 to June 2009 at 196.2* (44.9, 505.8), with its final trend from July 2009 to the time of data collection that demonstrated a less drastic but significant 6mPC at 2.6* (1.2, 4.1). 

As far as treatment and interventions were concerned, “prostate biopsy” demonstrated just one trend for the entirety of the study with a significant 6mPC at 0.7* (0.2, 1.2). “Prostatectomy” (compared to prostate radiation), demonstrated two trends, with the first demonstrating a negative trend of -1.7* (-2.2, -1.1) from January 2004 to December 2015, and then became positive starting from January 2016 with a significant 6mPC of 3.3* (1.4, 3.5). “Prostate Radiation” (compared to prostatectomy) had one consistent significant trend throughout the study with a 6mPC of 1.22* (0.6, 1.9). “Prostatectomy side effects” (compared to radiation side effects) also had one significant trend throughout the study, with a very positive 6mPC of 14.4* (9.5, 19.6). “Radiation side effects” (compared to prostatectomy side effects) also demonstrated a single positive 6mPC of 21.4* (13.1, 29.1) throughout the study, with no changes in slope noted.

## Discussion

As healthcare continues to incorporate the internet and electronic resources for providing care and educating patients, it is of no surprise that the internet has now become integral to understanding a prostate cancer diagnosis. Our study demonstrates that men are using the internet more each year, with all but two terms demonstrating a positive total average of 6mPC. Furthermore, we found that men were seeking additional information on procedures (i.e. prostate biopsy & prostatectomy) and treatments (radiation), as demonstrated by higher aSVI for all these searched terms. Monitoring search trends provides an opportunity for improvement in both patient care and satisfaction as well as insight into disease management in an evolving field.

PSA-specific trends

Understanding the significance and role of PSA screening in men continues to be confusing. We found that the internet is used to gain additional information about PSA and related terms such as “what is PSA” (aSVI 23.55), which continues to be searched at relatively high volumes as is demonstrated with positive trends and Joinpoint analysis throughout the study period (Figures 1, 2). With conflicting recommendations on PSA screening from different organizations and physicians, we hypothesize that men turn to the internet to seek clarification to better understand the significance of their specific PSA levels.

Treatment and geographic trends

Prostatectomy and radiation remain the two primary treatment options for men with locally advanced prostate cancer. Google searches for prostatectomy were stable throughout the study period with 6mPC at -0.2. However, it should be noted that there was a slight although significant uptick starting in January 2016. Prostate radiation also demonstrated a stable 6mPC at 1.22 throughout the study. In contrast, more men are enrolling in active surveillance, however search volumes remain relatively low. The lower search volumes for that term make trend analysis difficult to interpret for that term and it is unclear how or if the internet is being utilized for these men.

As far as geographic trends, the Eastern United States demonstrated a higher search volume than the Western, Southwest, or Midwest regions. Regarding the Eastern United States, the Northeast demonstrated higher volumes for general terms such as “prostate cancer”, “prostate cancer survival”, and “Gleason score”, while the Southeast demonstrated higher search volumes for terms associated with treatment or intervention such as “prostate biopsy”, “prostatectomy”, and “prostate radiation”. Previous studies have demonstrated that geographic location plays a significant role in prostate cancer treatment. One study demonstrated that surgical rates were highest in the West and lowest in the Northeast [10], a pattern that has been demonstrated on multiple occasions [11,12]. Unsurprisingly, race and ethnicity have also been shown to be associated with prostate cancer treatment, with those with higher socioeconomic status, white, and older demonstrating a higher likelihood of receiving radiation therapy, while minorities were more likely to enroll in active surveillance [13].

Quality of life

Quality of life concerns in prostate cancer treatment continue to be an important topic for physicians, patients, and their loved ones [14]. Both prostatectomy and prostate radiation carry significant side-effect profiles including incontinence and a decline in sexual function. Our study demonstrates that side effects are searched more each year, with notable positive 6mPCs' demonstrated on Joinpoint analysis (14.4 for prostatectomy side effects and 21.4 for radiation side effects). Educating men on prostate cancer is critical to helping them understand whether treatment options are within their personal goals and what they can expect following their treatment.

Previous studies demonstrated that nearly all men experience a significant decline in quality of life in the one to two years following treatment, regardless of treatment modality [14]. They also demonstrated that most individuals demonstrated a plateau from three to 10 years following the initial decline in quality of life. Given the importance of post-treatment quality of life and difficult treatment decisions, proper education on quality-of-life expectations is necessary.

Significant time points

Perhaps most interestingly, there were two-time points identified that demonstrated a deflection point for multiple search terms. The first was around 2007-2008 in which the terms “radiation side effects”, and "what is normal PSA?", "active surveillance", and "Gleason score" all had trend changes. All these terms except for “active surveillance” either changed from a positive trend to a negative trend or changed from a sharply positive trend to a much lower positive trend. The other deflection point was noted around 2014 with the terms “prostate cancer”, “prostatectomy”, and “prostate-specific antigen,” all changing from a slightly negative trend to a positive trend at this time. Unsurprisingly, these changes seem to align with guideline recommendation changes [15].

The benefits vs. harms of PCa screening have been controversial by way of screening guidelines, from various organizations, that have shifted and been revised since the advent of PSA screening. However, there were many growing concerns about PSA screening; regarding overdiagnosis, unnecessary biopsies, and over-treatment leading to untoward side effects. In 2008 and again in 2012, U.S. Preventive Services Task Force (USPSTF) issued a grade D recommendation against screening men >75 years old, consequently reducing Medicare coverage for PSA testing, and creating the narrative amongst primary care physicians to reject PSA tests. Following these guidelines, PSA testing declined in the U.S. by 25-30%, which is congruent with our data that show negative trends in 2008 for search terms such as “what is normal PSA ” and “Gleason score.”

The result of these recommendation guidelines that went against PSA screening unfortunately gave way to increasing high-grade disease at diagnosis. Studies then supported that PSA screening, within an organized program, was more effective [16]. In 2017, Cancer Intervention & Surveillance Network (CISNET) re-evaluated results from the same randomized controlled trials (RCTs) from which the UPSTSF had based their recommendations, revealing incorrectly reported evidence and greater reductions in PCa mortality than reported [17]. Given the variation in recommendations and likely variation from physicians, it is clear that many individuals may have been confused about PSA screening and its clinical significance. Unsurprisingly, our study demonstrates the use of the internet to gain additional information about PSA generally. Our data showed PSA and related terms such as “what is PSA”, and “what is normal PSA” to be positive throughout the study period, and are still being searched more each year at a relatively high volume.

Limitations

The study is not without limitations, and interpretation should be made thoughtfully. These limitations include the anonymous nature of the data which prevents specific analysis of sub-population groups. Age is not included in the data. Given the older age of the typical man diagnosed with prostate cancer, the inability to control for age limits some conclusions. It is also unknown if it was the patient, a family member, or other non-related individuals (health care professionals, etc) who was doing these searches. The search terms also only included English search terms, and any non-English languages were not captured. This may lead to an under-representation of certain populations or areas.

## Conclusions

The internet continues to be a growing part of the dynamics of diagnosing and treating locally advanced prostate cancer. We found that individuals are particularly interested in information pertaining to PSA as well as procedures and interventions affecting the quality of life. This suggests that physicians and other providers should continue to focus on these areas for patient education. Additionally, we recommend providing accessible and reliable internet resources to help guide patients.
